# Anticorrosion and Cytocompatibility Assessment of Graphene-Doped Hybrid Silica and Plasma Electrolytic Oxidation Coatings for Biomedical Applications

**DOI:** 10.1021/acsbiomaterials.1c00326

**Published:** 2021-11-08

**Authors:** Juan P. Fernández-Hernán, Antonio J. López, Belén Torres, Enrique Martínez-Campos, Endzhe Matykina, Joaquín Rams

**Affiliations:** † Departamento de Matemática Aplicada, Ciencia e Ingeniería de Materiales y Tecnología Electrónica, ESCET, Universidad Rey Juan Carlos, C/Tulipán s/n, 28933 Móstoles, Spain; ‡ Instituto de estudios biofuncionales, ICTP-CSIC, Universidad Complutense, Paseo Juan XXIII No 1, 28045 Madrid, Spain; § Departamento de Ingeniería Química y de Materiales, Facultad de Ciencias Químicas, Universidad Complutense, 28040 Madrid, Spain

**Keywords:** magnesium, sol−gel, plasma electrolytic oxidation, GNP, coatings, corrosion, cytocompatibility

## Abstract

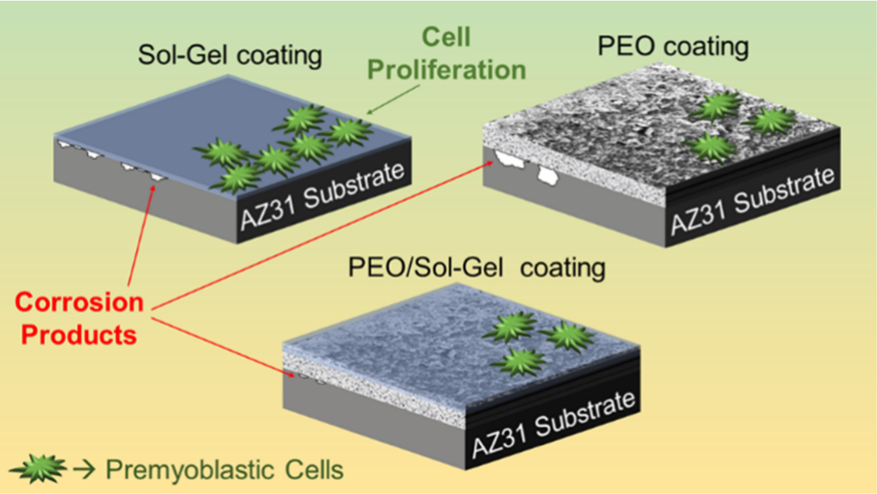

Magnesium AZ31 alloy substrates were coated with different coatings, including sol–gel silica-reinforced with graphene nanoplatelets, sol–gel silica, plasma electrolytic oxidation (PEO), and combinations of them, to improve cytocompatibility and control the corrosion rate. Electrochemical corrosion tests, as well as hydrogen evolution tests, were carried out using Hanks’ solution as the electrolyte to assess the anticorrosion behavior of the different coating systems in a simulated body fluid. Preliminary cytocompatibility assessment of the different coating systems was carried out by measuring the metabolic activity, deoxyribonucleic acid quantification, and the cell growth of premyoblastic C2C12-GFP cell cultures on the surface of the different coating systems. Anticorrosion behavior and cytocompatibility were improved with the application of the different coating systems. The use of combined PEO + SG and PEO + SG/GNP coatings significantly decreased the degradation of the specimens. The monolayer sol–gel coatings, with and without GNPs, presented the best cytocompatibility improvement.

## 1 Introduction

The use of magnesium alloys for biomedical applications, especially for the manufacture of resorbable implants for bone fracture treatments, has aroused interest over the last years due to their outstanding properties.^1−6^ Magnesium is a biocompatible and bioresorbable material, and the Mg^2+^ cation is present in a wide range of biochemical and physiological processes and is an osteoconductive material that could help promote the growth of bone tissue. Moreover, magnesium is the lightest of the structural metals, with density and stiffness values close to that of natural bone tissue. Thus, the use of magnesium-based implants could help to avoid or decrease the stress shielding effect, which promotes osteopenia, that is, the resorption of the bone tissue that is not exposed to the normal mechanical loads due to the existence of a metal implant with a higher stiffness value, which supports the mechanical load.^7,8^

However, the high reactivity of this element is the main reason that magnesium is not widespread as the main base material for temporary bioresorbable implants. Magnesium alloys are prone to suffer from corrosion processes,^9^ especially when specific metallic impurities are present in the alloy or when these alloys are exposed to chloride-containing electrolytes. In general, the corrosion process of magnesium alloys is initiated by localized corrosion. This corrosion type can appear as pitting corrosion in the α-Mg and Mg_17_Al_12_ interphases of Mg–Al alloys, like in the case of AZ31 alloys. Filiform corrosion can also occur as a localized corrosion process, especially in the case of Mg alloys treated with protective coatings. There are two factors responsible for the low corrosion resistance of magnesium alloys. The first factor is the poor stability of the hydroxide passivation coating that forms on magnesium alloys. Contrary to other passivation layers naturally generated, this oxide layer provides low protection for the underlying substrate. The other factor is the galvanic corrosion that occurs due to the presence of intermetallic phases or impurities in the alloy.^10,11^

During the corrosion process, hydrogen is generated in the cathodic reaction. Once implanted, if the hydrogen evolution rate is higher than the capacity of the body to assimilate and eliminate it, hydrogen bubbles can accumulate and modify some parameters like pH,^12,13^ affecting the tissues surrounding the implant, which would be a risk for the patient.^14^ Moreover, if the degradation ratio of the bioresorbable magnesium implant is faster than the healing ratio of the natural bone, the early loss of the mechanical integrity can cause the failure of the implant,^15^ affecting the healing process of the fractured bone and making it necessary for a second surgery, with the subsequent risk for the patient.

To overcome the main drawback of the magnesium-based implants, several strategies can be followed to improve their resistance against corrosion and to control the degradation rate of magnesium-based bioresorbable implants, such as cathodic protection, alloy modification, surface modification, coatings, and so forth.^16^ Among these strategies, the application of coatings has been demonstrated to be an effective way to protect the metallic substrates from aggressive media, improving the corrosion resistance of the coated materials.^17^

Moreover, the use of different coating strategies can be found in the literature to improve the biocompatibility properties of the coated substrates. The use of sol–gel coatings or plasma electrolytic coatings is widespread to improve the biocompatibility of metals used for implants, enhancing the cellular adhesion and proliferation on their surfaces.^18−22^

In addition, the properties of the coatings can be enhanced by adding different substances or elements during the synthesis process. For example, in the case of coatings generated by the sol–gel method or by plasma electrolytic oxidation (PEO), different fillers and dopant substances, like corrosion inhibitors, nanoparticles, growth factors, antibiotics, collagen, and so forth, can be added to improve specific properties like improved anticorrosion, higher mechanical resistance, biocompatibility, or antibacterial behavior.^23−31^

Regarding nanoparticles, different studies can be found in the literature regarding graphene nanoplatelets (GNPs) being used as nanofillers for enhancing the corrosion and wear protection of coatings deposited on metallic substrates.^32,33^ However, the biocompatibility of GNPs generates controversy. Some studies claim that the interaction between GNPs and cells leads to cell damage. Due to their morphology, GNPs can damage the plasma membrane and accumulate inside the cell, interacting with cell organelles and promoting oxidative stress, which harms the cell. However, these results depend on the cell type and concentration of nanoparticles. At a very low concentration, cells can overcome the possible damages caused by the nanoparticles.^34−36^

In this research, two different methods were used to generate biocompatible anticorrosion protective coatings. First, the sol–gel synthesis route was followed to generate compact and homogeneous hybrid silica coatings from two silicon alkoxides.^32,37−40^ In this case, the samples were coated by the dip-coating method. Second, coatings were generated through the PEO method.^19,41,42^ In this case, the coating grows on the surface of the metallic substrate immersed in an electrolyte due to microdischarges occurring on the surface of the sample, generating an anodic oxide layer that contains elements present in the electrolyte. Furthermore, in this study, the two different coating methods were combined to generate a bilayer coating system, consisting of a first layer generated by PEO and a second sol–gel layer applied by dip-coating. These bilayer systems are intended to overcome the handicap of the PEO method because these coatings are intrinsically porous due to the gas evolution that occurs on the surface of the substrate while the anodic oxide coating is growing. This porosity is an important concern because interconnected pores can create direct pathways, connecting the surface of the metallic substrate with the aggressive medium, decreasing the protective barrier effect of the PEO coating.^43^ However, by combining PEO with sol–gel coatings, the pores in the PEO coating can be sealed by the sol–gel, which will increase the protective anticorrosion properties of this bilayer system.^44^

Once implanted in the body, the biological interactions will take place between the coating and the surrounding cellular tissues. Therefore, the biocompatibility behavior of the different coating systems is a big concern and must be assessed. Different factors play an important role in the biocompatibility of a material, such as composition, roughness, hydrophobicity, and so forth.^45^ Previous studies show that silica-based glasses are biocompatible materials.^46^ PEO coatings have also been studied, and biocompatibility properties were found in this kind of coatings.^47^ Cellular cultures can be performed on the surface of the different coating systems to assess the cellular adhesion on these materials and their cytocompatibility.

This research aims to generate different cytocompatible coating systems, not to avoid corrosion but to control and decrease the degradation rate of AZ31 magnesium alloy substrates, when they are immersed in a simulated biological environment, to obtain feasible protective coatings for interesting magnesium alloys for biomedical usage. Moreover, these coating systems are intended to improve cytocompatibility by enhancing cell adhesion and proliferation over their surfaces. To achieve these goals, two different coating methods, sol–gel and PEO were used to generate monolayer and combined bilayer coating systems. Different techniques such as linear polarization resistance, anodic–cathodic polarization, and hydrogen evolution tests were carried out to assess the protection against corrosion of the different coating systems immersed in simulated body fluid (SBF) medium (Hanks’ solution). Moreover, the cytocompatibility of the different coatings was assessed by measuring the metabolic activity and cell viability and through DNA quantitation of premyoblastic C2C12-GFP cells cultured on the different coating systems. The results of these experiments show that both, monolayer sol–gel coatings and combined PEO/sol–gel coatings, decreased the corrosion rate and improved the cytocompatibility, compared with the bare AZ31 magnesium substrates.

## 2 Experiment Section

### 2.1 Substrate Material

AZ31 magnesium alloy plates were provided by Magnesium Elektron, with a composition in wt % of 2.9 Al, 0.75 Zn, 0.29 Mn, 0.01 Si, <0.005 Ca, 0.004 Fe, 0.0013 Ni, <0.0005 Cu, and balance Mg. These plates were cut to obtain the substrate samples of 15 × 15 × 2.5 mm^3^ which, previous to coating, were ground with SiC 1200 grit papers, degreased in an ultrasonic isopropanol bath for 10 min, and air-dried.

### 2.2 Coating Generation

**Figure 1:**
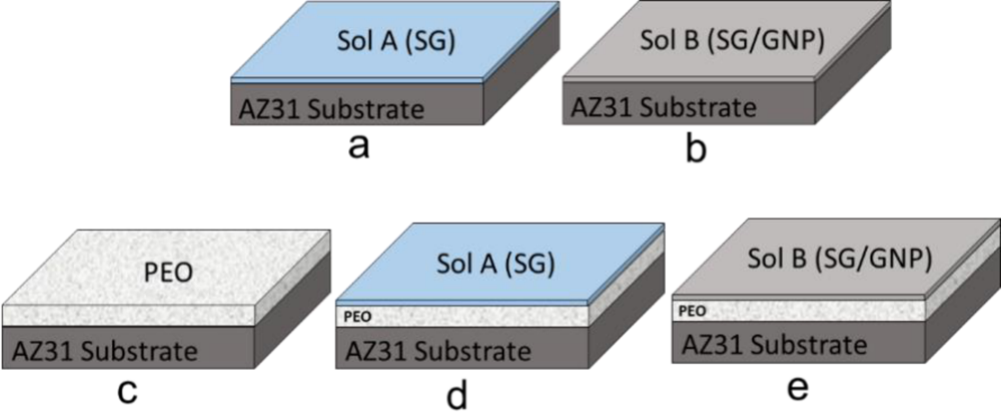
(a) Monolayer sol–gel coating. (b) Monolayer sol–gel coating doped with functionalized GNPs. (c) Monolayer PEO coating. (d) Combined PEO and sol–gel bilayer coating. (e) Combined PEO and sol–gel doped with GNP bilayer coating.

In this study, five different coating configurations were developed (Figure 1). The first and the second systems consisted of monolayer hybrid silica coatings generated from two different hybrid sol–gel (sol A and sol B), deposited on the surface of the AZ31 substrates by dip-coating (Figure 1a,b). Both sol–gel (sol A and sol B) were synthesized from two silicon alkoxides, tetraethyl orthosilicate [TEOS; Si(C_2_H_5_O)_4_], and methyl-triethoxysilane [MTES; CH_3_–Si(C_2_H_5_O)_3_] in a molar fraction of 40% TEOS and 60% MTES. The mixture of these precursors was stirred for 30 min at room temperature and then diluted in isopropanol and 0.1 M HCl acidulated H_2_O in a molar ratio of 1 mol of the mixture of precursors, 5 mol of isopropanol, and 10 mol of acidulated H_2_O. In addition, sol B was doped with 0.005 wt % grade 4 −COOH functionalized GNPs (COOH–GNPs), with a thickness value lower than 4 nm and 1–2 μm wide, provided by Cheap Tubes Inc. The final mixtures for both, sol A and sol B, were then stirred for 2 h at room temperature. After this time, the sols were left to stand for 30 min to let the hydrolysis and polycondensation reactions be completed. Table 1 lists the composition of the different sols. The composition of the sol–gel was selected in previous laboratory tests.^32^

**Table 1:** Composition of the Two Different Sol–Gel Used to Generate the Coatings

sol–gel	composition (molar ratio = 1:5:10)	nanocharges
sol A	40% TEOS/60% MTES/isopropanol/0.1 M HCl–H_2_O	
sol B	40% TEOS/60% MTES/isopropanol/0.1 M HCl–H_2_O	0.005 wt % COOH–GNPs

Once the sols were synthesized, the AZ31 substrates were coated by dip-coating. In this process, the samples were immersed in the corresponding sol–gel for 1 min. After this time, the samples were extracted from the sol–gel with a controlled withdrawal speed of 35 cm/min. Then, a low temperature and long-lasting thermal treatment were applied, consisting of a drying treatment at 100 °C for 24 h, followed by a sintering treatment at 200 °C for 24 h, to avoid the thermal deterioration of the magnesium substrates. The final GNP concentration in sol–gel coatings generated from sol B was 0.045 wt %, due to the evaporation of the liquid phases during the drying process.

With this first coating technique, two different monolayer coatings were developed: monolayer sol–gel coating without nanocharges (SG) (Figure 1a) and monolayer sol–gel coating doped with 0.045 wt % functionalized GNPs (SG/GNP) (Figure 1b).

The third coating configuration consisted of a monolayer oxide coating, grown on the surface of the AZ31 substrates by PEO (Figure 1c). The AZ31 alloy samples served as anodes with a total exposed area to the electrolyte of about 5.7 cm^2^. A 316L stainless steel cylindrical mesh was used as the cathode. The composition of the electrolyte used in this coating process is shown in Table 2. The PEO layers were formed in AC mode during 320 s at a controlled temperature of 20 °C by the application of a square waveform with an rsm voltage of 480 V (+430 V, −50 V) and a limiting current density of 138 mA/cm^2^ at a frequency of 50 Hz. These conditions, and the composition of the electrolyte, were selected in previous laboratory tests.^48^

**Table 2:** Electrolyte Composition for the PEO Coating Process

compound	concentration (g/L)
Na_3_PO_4_·12H_2_O	10
NaF	8
KOH	1
CaO	2.9

Finally, two different bilayer coating systems were developed. The first one consisted of a PEO monolayer coating combined with a second layer of sol–gel from sol A, without nanocharges, named PEO + SG (Figure 1d). The last coating system consisted of a combination of a monolayer PEO coating with a second layer of sol–gel from sol B, doped with a final concentration of 0.045 wt % COOH–GNPs, named PEO + SG/GNP (Figure 1e).

### 2.3 Coating Characterization

For the characterization of the different coating systems, a scanning electron microscope (SEM Hitachi, S-3400N, 15 kV acceleration voltage, 10 mm working distance, secondary and backscattered electrons imaging) was used to evaluate the thickness and homogeneity of the coatings and to determine the presence of cracks after the coating process. EDS–SEM tests were also carried out (Bruker AXS XFlash detector 5010) to determine the composition of the different coating systems and to assess the interaction between the PEO and the sol–gel coatings in the bilayer systems.

A surface profilometer (Mitutoyo SJ-210) was also used to determine the roughness of the different sol–gel coating systems and compare them with the roughness of the bare AZ31 substrate. The ISO1997 standard was used, and cutoff values of λc = 2.5 mm for PEO samples and λc = 0.8 mm for the other samples were used.

Finally, contact angle tests were carried out to determine the hydrophobicity of the different sol–gel coating systems, using distilled water as the liquid phase and a goniometer (RAMÉ-HART 200-F1) to take photographs of the drops on the surface of the samples and to measure the contact angles.

### 2.4 Corrosion Tests

Linear polarization resistance tests were carried out using a Metrohm Autolab PGSTAT302N potentiostat. The samples were immersed in Hanks’ solution (pH = 7) in a three-electrode cell configuration, using a Ag/AgCl reference electrode, a graphite rod as the counter electrode, and the sample as the working electrode. The applied potential was ±10 mV around the corrosion potential (*E*_corr_) with a scanning rate of 1 mV/s. After a stabilization time (1 h), polarization resistance (*R*_p_) measurements were carried out after 1 h of immersion of the samples in Hanks’ solution, and then every 24 h until a total immersion time of 168 h. The *R*_p_ values were obtained from the slope of the linear region in the *E* (V) versus *I* (A) plot obtained during the test.

Electrochemical anodic–cathodic polarization tests were developed for two different immersion times, 1 and 24 h, both using Hanks’ solution as the electrolyte and, like in the case of the linear polarization resistance tests, using a three-electrode cell configuration. After the stabilization time (1 h), the tests were carried out using a scanning range of 1000 mV (−400/+600 mV) around the corrosion potential (*E*_corr_), with a scanning rate of 1 mV/s. From these tests, current density values were calculated by following the method reported by Stern and Geary,^49,50^ from the polarization resistance (*R*_p_) values and the proportionality constant *B*, eq 1. The value of this constant depends on the slopes of the anodic (*b*_a_) and cathodic (*b*_c_) curves of the anodic–cathodic diagrams. |*b*_a_| and |*b*_c_| were calculated directly from the Tafel plots, using software Nova 2.1 provided with the potentiostat used in these tests. The relation between these parameters is shown in eqs 1 and 2.
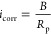
1
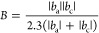
2

Magnesium alloys present an electrochemical phenomenon known as the negative difference effect (NDE), which makes it difficult to reliably assess the corrosion rate of magnesium and its alloys by electrochemical tests.^10,51^ On the other hand, the estimation of the degradation rate by weight loss measurements only offers information at the end of the test. Thus, complementary hydrogen evolution tests are usually carried out for a better assessment of the corrosion behavior. This method, described by Song et al.,^9,52^ is an easy and accurate way to assess the corrosion rate of magnesium and its alloys, based on the overall corrosion reaction of magnesium in aqueous solutions.
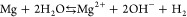
3

From eq 3, it is possible to estimate the amount of corroded magnesium by measuring the evolved hydrogen from the corroding sample because 1 mol of evolved H_2_ corresponds with the degradation of 1 mol of magnesium.

**Figure 2:**
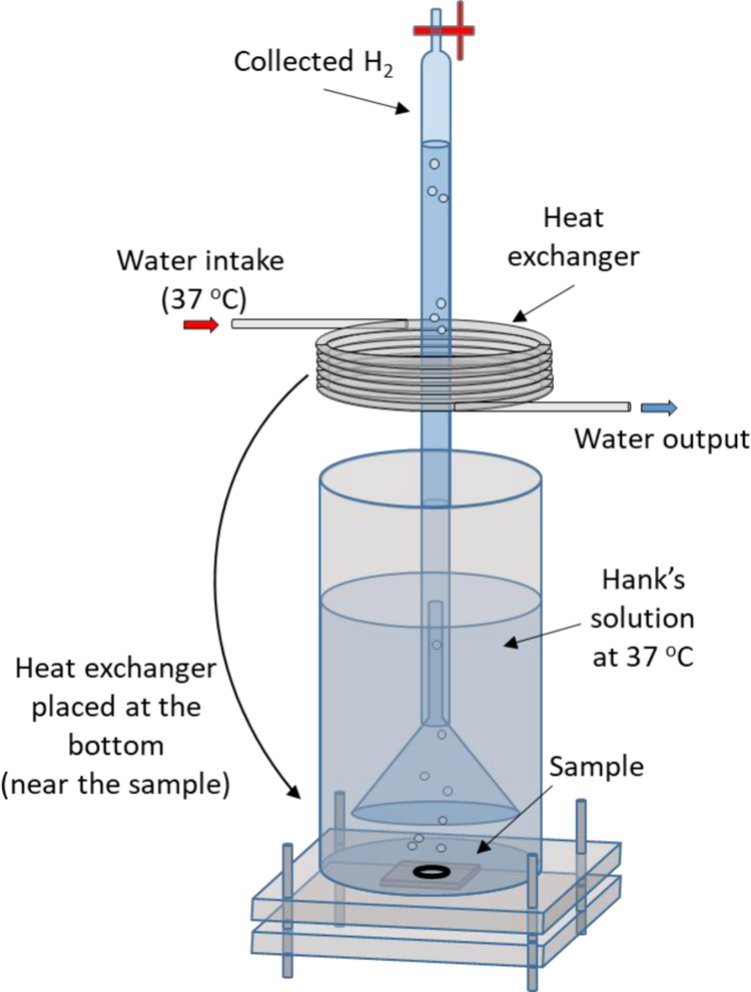
Setup used to estimate the hydrogen evolution under simulated biological conditions.

The hydrogen evolution tests were carried out using the setup shown in Figure 2, with an exposed sample area to Hanks’ solution of 0.75 cm^2^. The volume to exposed surface ratio of Hanks’ solution was 173 mL/cm^2^. The solution was not renovated during the experiment. A heat exchanger was added to the original setup to maintain the temperature of the electrolyte at 37 °C to simulate the biological temperature conditions. The volume of H_2_ was measured for 168 h at intervals of 24 h.

One simple way to assess the in vitro degradation is to calculate the corrosion rate for each sample. This parameter can be obtained from the values of evolved hydrogen.^53^Equation 4 shows the relation between the weight loss in mg/cm^2^/d and the average corrosion rate in mm/y.
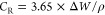
4In this equation, Δ*W* represents the weight loss of the corroded sample and ρ is the density of the material. For magnesium, this parameter takes the value of 1.74 g/cm^3^. In the corrosion reaction of magnesium, one molecule of hydrogen is evolved from the reaction of one atom of magnesium. Thus, the relation between the weight loss and the evolved hydrogen (*V*_H_) in mL/cm^2^/d is shown in eq 5.
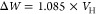
5Replacing Δ*W* from eq 5 in eq 4, the corrosion rate can be obtained as a function of the volume of evolved hydrogen from the corroded sample. For a magnesium alloy, this relation is shown in eq 6.
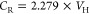
6

Because the coating systems are intended to protect and control the degradation rate of the magnesium substrate for medical treatments, an in vitro experiment was developed to assess the degradation of the AZ31 substrates coated with the different coating systems. The samples were immersed for 1 week in Hanks’ solution (pH = 7) at 37 °C, with their whole surface exposed to the electrolyte. During this time, pH measurements (CRISON Basic 20) were made regularly, as well as micrographs at the end of the experimentation time, to evaluate the protection provided by the different coating systems.

### 2.5 Preparation for Cytocompatibility Tests

Before cytocompatibility tests, all the coated samples were sterilized following the next sequence: the samples were immersed in 70% ethanol for 10 min and repeated three times. Then, the samples were rinsed three times in PBS solution for 10 min each. The samples were placed in multiwell plates with 1 mL of PBS and sterilized with ultraviolet germicidal irradiation (UVGI) for 40 min. After the UVGI, the samples were rinsed in a PBS bath for 10 min. Then, the samples were covered with DMEM (GIBCO) solution for 10 min. Finally, the samples were immersed in 1 mL of DMEM supplemented with 10% FBS (Thermo Scientific) plus antibiotics (100 U mL^–1^ penicillin and 100 μg mL^–1^ streptomycin sulfate, Sigma-Aldrich). The last step in the preparation phase was to store the samples immersed in the completed culture medium at 37 °C for 24 h.

### 2.6 Cell Culture

The cell seeding was performed using a mouse pre-myoblast cell line, the C2C12-GFP (ATCC CRL-1772). Green fluorescent protein (GFP) was expressed due to a previous lentivirus infection of the C2C12 cell line. The C2C12-GFP cells were seeded on the different coating systems with a cell concentration of 4 × 10^4^ cells/cm^2^, covered with 2 mL of complete medium, and incubated at 37 °C.

### 2.7 Cytocompatibility Assessment

Inverted fluorescence microscopy (Olympus IX51) was used to evaluate the culture growth and the cell adhesion on the surface of the different coating systems. Micrographs were taken at 24, 48, 72, and 168 h after the cell seeding (FITC filter λ_ex_/λ_em_ = 490/525 nm).

Complementary to fluorescence microscopy, three different tests were carried out to evaluate biological behavior over the different coating systems:

(i) To assess the cell population proliferating over surfaces with different coating systems, 200 μL of trypsin was added to the different cultures to detach the cells and obtain a homogeneous cell suspension. After 15 min, 200 μL of DMEM was added to the previous volume of trypsin to stop the protease reaction. 10 μL of the final trypsin/DMEM mixture containing the cells detached from the surface of the different samples was put in a Neubauer hemocytometer to count the viable cells after 168 h of the experiment.
(ii) In addition, the metabolic activity of the cellular cultures was evaluated by alamarBlue tests^54^ (Thermo Fisher) carried out after 72 and 168 h of culture time. Using the reducing power of cell machinery, this nontoxic technique allows for the quantification of mitochondrial activity in living cells. Three specimens were evaluated for each culture time. In this method, the alamarBlue dye was added to the culture medium of each sample (10% of the volume of the culture medium). Then, the samples were incubated for 90 min. Finally, the fluorescence for each sample was measured using a microplate reader (BioTek, Synergy HT).
(iii) DNA quantitation was carried out using the blue-fluorescent Hoechst 33258 nucleic acid stain, following the manufacturer’s protocol^55^ (Thermo Fisher, FluoReporter). This assay was developed after 168 h of culture time. The fluorescence for each sample was measured using a microplate reader (BioTek, Synergy HT).

Statistical analysis was carried out for the metabolic activity, DNA quantitation, and cell growth tests (mean value ± standard deviation), with a confidence interval of 95% (*p* < 0.05) consisting of one-way ANOVA and Tukey’s post-hoc tests.

## 3 Results and Discussion

### 3.1 Coating Characterization

**Figure 3:**
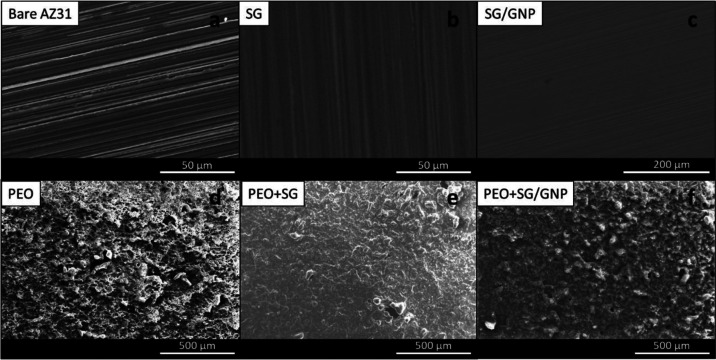
Plain view of the surface of the bare substrate (a) and the different coating systems (b–f) on the AZ31 substrates.

Figure 3 shows the plain view micrographs of the surfaces of the different conditions. In the bare substrate, as shown in Figure 3a, the grinding lines are visible. In the case of SG and SG/GNP coating systems, as shown in Figure 3b,c, respectively, the grinding lines are still visible below the sol–gel coatings, due to the low thickness values of these coatings and because they replicate the substrate surface roughness. However, no porosity, defects, or cracks were visible on the coating surfaces, indicating that the coatings were consolidated on the substrate and thick enough to not affect the structural integrity by the roughness of the substrates. Figure 3d shows the surface of the PEO coating system; in this case, due to the nature of the PEO process, the surface appears rough and large pores are distributed on the surface. Figure 3e,f shows how the combination of PEO and sol–gel coating systems works. In this case, for both PEO + SG and PEO + SG/GNP coating systems, it is possible to observe that sol–gel coatings cover the irregularities and seal the pores of the underlying PEO coating, decreasing the roughness of the final coating and homogenizing the surface finishing compared with the PEO coating system. However, some of the pores and irregularities are too large to be completely covered by sol–gel; thus, some defects remain in these combined coatings.

**Figure 4:**
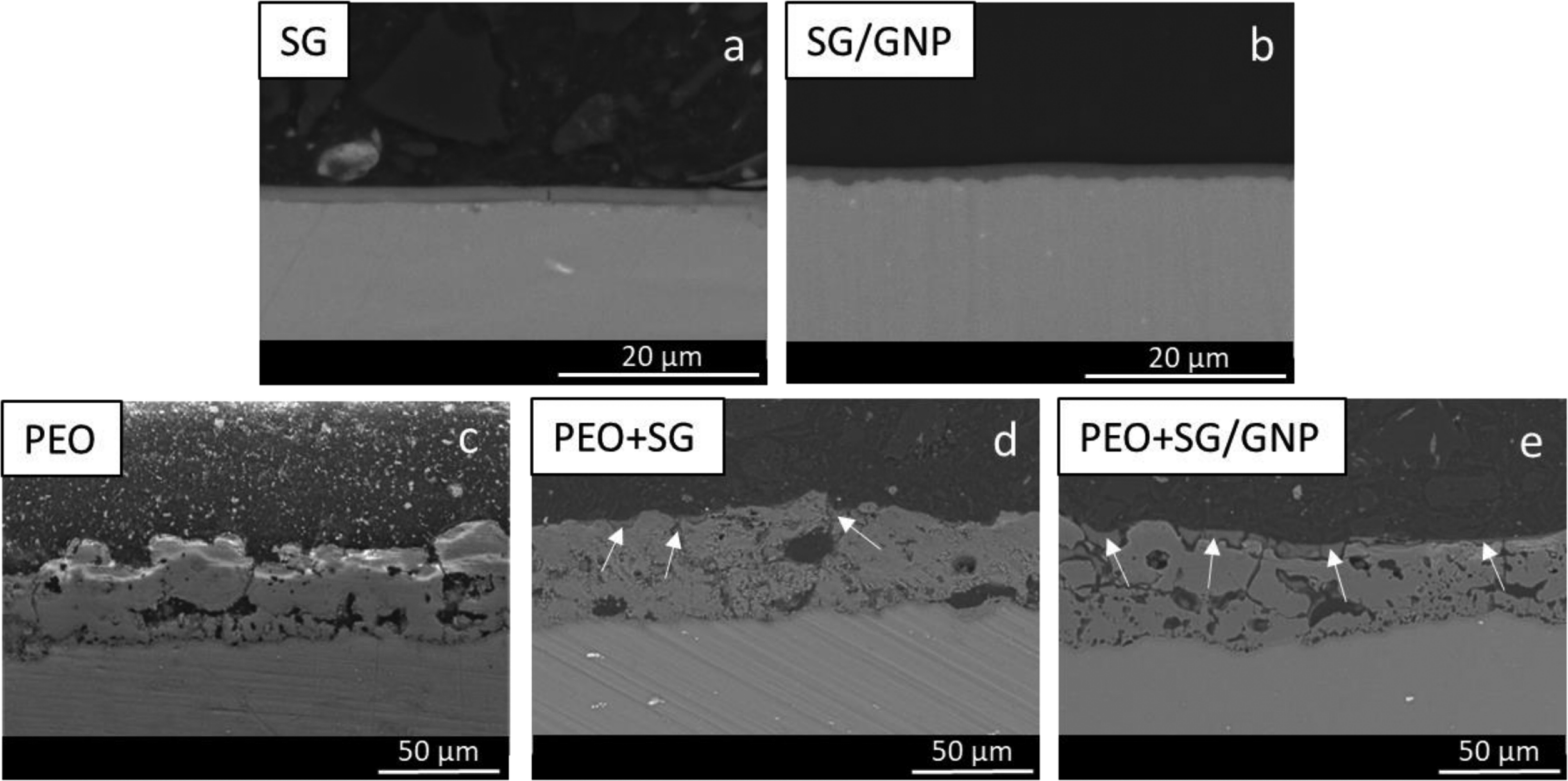
Backscattered electron micrographs of the cross-sectional view of the different coating systems on the AZ31 substrates.

**Figure 5:**
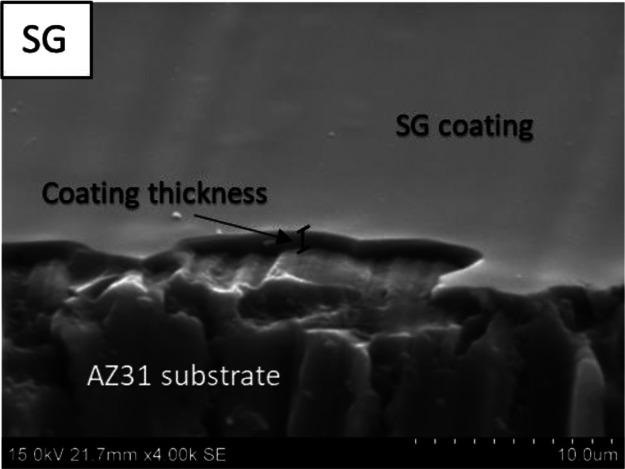
60° tilted view of the SG monolayer coating system on the AZ31 substrate.

Looking at the cross section of micrographs of the different coating systems (Figure 4), it is possible to assess the thickness and the inner structure of these coatings. The application of both SG and SG/GNP coating systems, as shown in Figure 4a,b, respectively, leads to materials with decreased roughness values, compared with the bare substrate. The sol–gel coatings follow the surface morphology of the substrates in the substrate-coating interface, but the outer coating surface tends to be smoother and homogeneous. As in the case of plain view micrographs, no cracks or defects are significant for these coating systems. The coatings adhere well to the surface of the AZ31 substrates. Sometimes, small cracks appear in these coatings. However, these cracks appeared during the preparation process of the samples for SEM assessment, which implies cutting, grinding, and polishing processes. The 60° tilted view of the sol–gel coating, as shown in Figure 5, reveals that no cracks or defects are present after the deposition of these coatings, providing good isolation and protection for the AZ31 substrates. The images show that monolayer sol–gel coatings with and without GNPs reach thickness values around 1.6 μm.

The cross section of micrographs of PEO coatings, as shown in Figure 4c, reveals that these coatings are thicker than monolayer sol–gel coatings, reaching thickness values around 35 μm. Higher thickness values of the coatings could provide better protective properties. However, as it is shown in the micrographs, PEO coatings are intrinsically porous because the coatings grow under dielectric breakdown conditions.^56^ The existence of internal pore interconnections can generate pathways that connect the surface of the substrate with the external aggressive medium, resulting in a decrease in the protective properties of these coatings. The combination of PEO and sol–gel coatings can help to overcome this drawback of the PEO coatings. Figure 4d,e shows how the sol–gel covers the pores and defects of the surface of the PEO + SG and PEO + SG/GNP coating systems, decreasing the porosity and the presence of direct pathways that could connect the aggressive medium with the surface of the magnesium substrates. The arrows mark the places where the sol–gel coating covers and seals the surface defects and pores of the underlying PEO coating.

**Figure 6:**
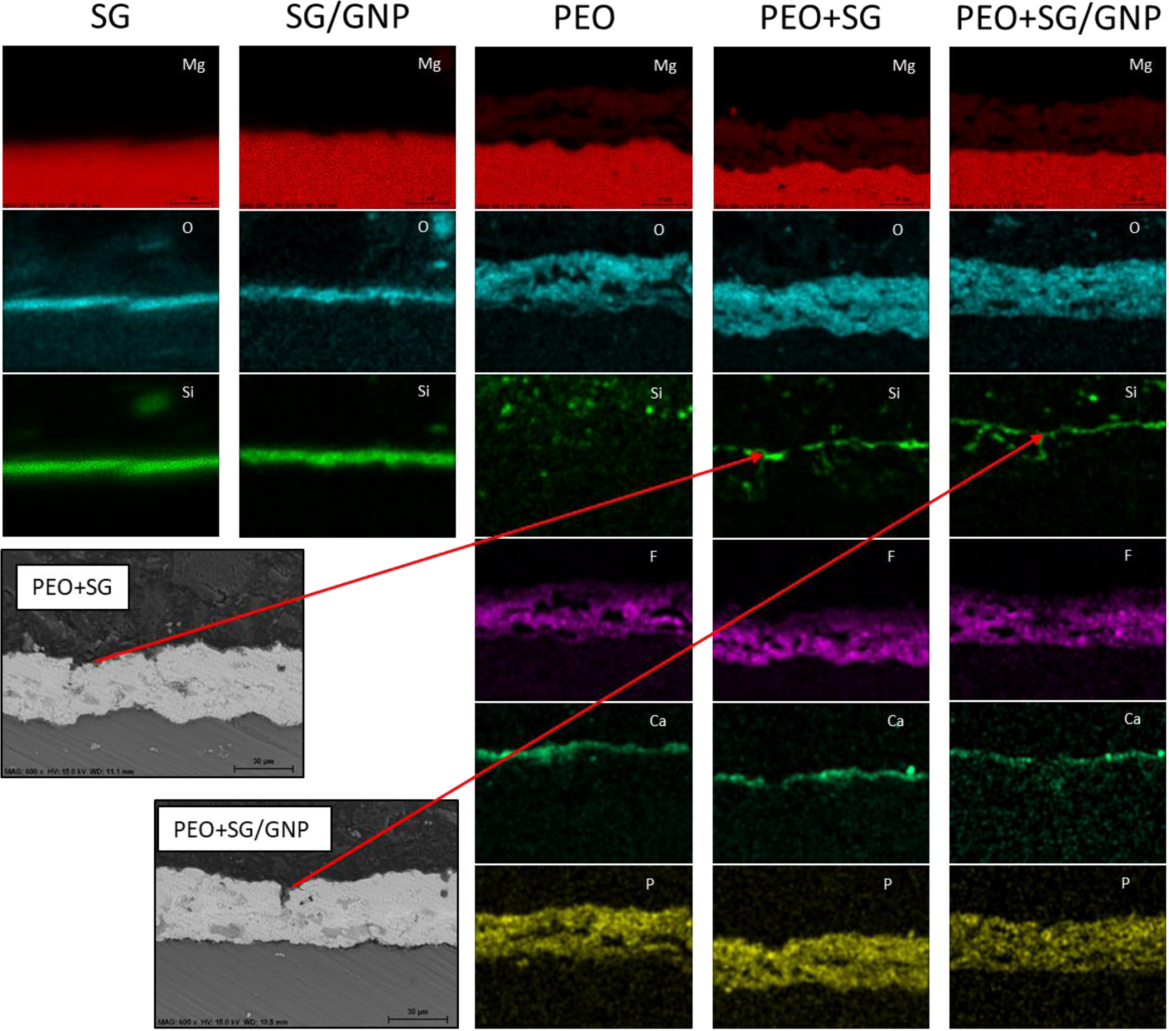
EDS–SEM analysis of the different coating systems. Silicon from the sol–gel coating is observed in the PEO + SG and PEO + SG/GNP coating systems, infiltrated in the pores of the PEO coatings.

As shown in Figure 6, EDS images show the distribution of the elements present in the coatings. For both SG and SG/GNP coating systems, silicon and oxygen are shown, indicating the presence of the silica coatings. The EDS images of PEO coatings show how the elements of the electrolyte are distributed in the coating. Oxygen, fluorine, and phosphorus are distributed homogeneously in the coating. However, calcium concentrates near the surface of the PEO coating. In the case of PEO + SG and PEO + SG/GNP, silicon is found creating a noncontinuous coating that fills the pores and defects of the surface of the underlying PEO coatings, blocking possible pathways from the surface of the substrate to the aggressive external medium.

**Figure 7:**
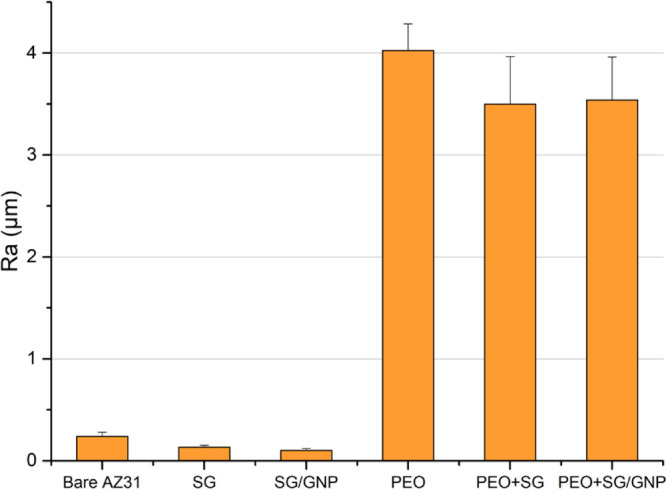
Roughness values of the surface of the different coating systems.

As previously exposed, the different coating systems result in different roughness values that could influence the cytocompatibility of the coatings. The roughness values for the different conditions are shown in Figure 7. The bare substrate ground with SiC 1200 grit paper has a mean roughness value (*R*_a_) of 0.24 μm. The deposition of sol–gel coatings on the surface of the AZ31 substrates leads to a significant decrease of the mean roughness values, which are 0.13 and 0.10 μm, respectively, for the SG and SG/GNP coating systems. The same behavior is observed for the combined PEO/sol–gel coating systems, where the application of the sol–gel coating on the PEO surface allows to obtain mean roughness values of 3.50 and 3.54 μm, respectively, which are lower than the mean value of the monolayer PEO coating, that is 4.02 μm. These results are consistent with the information extracted from the SEM micrographs, as shown in Figures 3 and 4.

**Figure 8:**
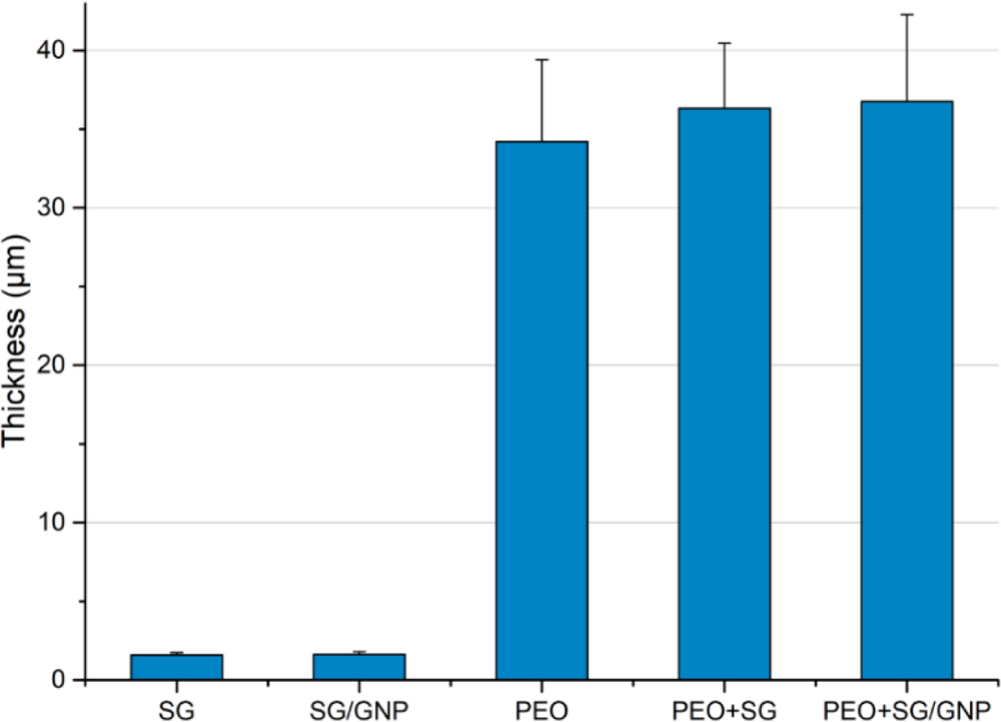
Coating thickness values of the different coating systems.

Figure 8 shows the thickness values of the different coating systems. As seen in the cross section of micrographs of the coatings, PEO coatings are much thicker than sol–gel monolayer coating systems. PEO + SG and PEO + SG/GNP coating systems with 36.3 and 36.7 μm, respectively, have the highest mean value of thickness, but there are no significant differences compared with the PEO coating, with a mean thickness value of 34.2 μm.

The SG and SG/GNP coating systems present mean thickness values of 1.5 and 1.6 μm, respectively, which are 1 order of magnitude lower compared with the values of the PEO coatings.

**Figure 9:**
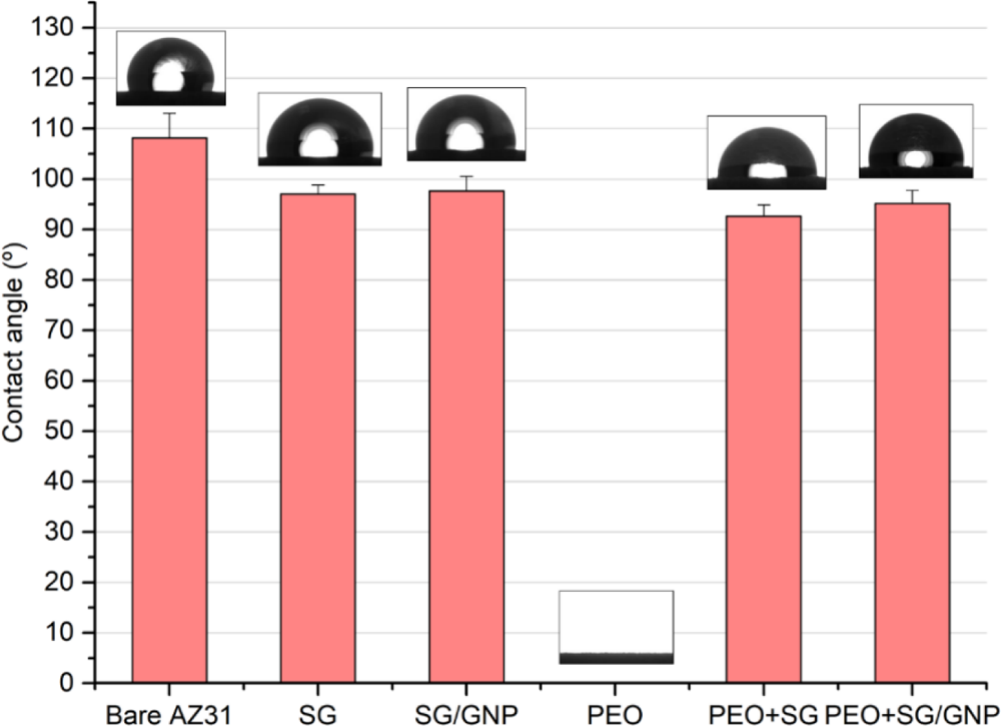
Contact angle values of the distilled water drop on the surface of the different coating systems.

The hydrophobicity of the different coating configurations is shown in Figure 9. The contact angle in the case of the bare substrate, 109.8°, is significantly higher compared with all the coating systems. It was impossible to assess the contact angle for the PEO coating. Due to the high porosity of this condition, the drop was absorbed as seen in the goniometer image for the PEO coating system in Figure 9. As shown in Figures 3 and 4, the sol–gel coating in PEO + SG and PEO + SG/GNP coating systems causes a decrease in the surface roughness and fills the pores of the PEO coatings, making it possible to measure the contact angle on these surfaces.

The values of the contact angle of PEO + SG and PEO + SG/GNP systems were 92.7 and 95.2°, respectively, which means a reduction in the contact angle of 16 and 13%, compared with the values of the bare substrate. In the case of the SG and SG/GNP coating systems, the contact angle values were 97.0 and 97.7°, respectively, achieving a reduction in the contact angle of 12 and 11%, compared with the values of the bare substrate. The mean values for the monolayer sol–gel coating systems are slightly higher than the values of the PEO + SG and PEO + SG/GNP coating systems, but no significant differences were found between these four conditions.

### 3.2 Corrosion Behavior Assessment

**Figure 10:**
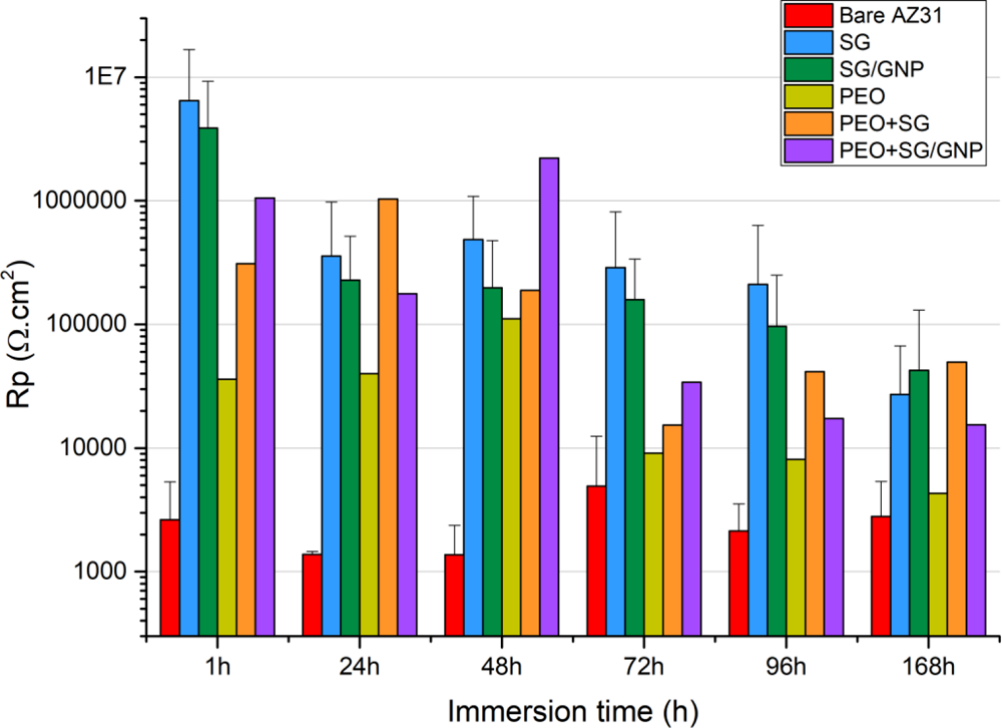
Linear polarization resistance values for the different coating systems immersed in Hanks’ solution up to 168 h.

The polarization resistance (*R*_p_) values of the bare substrate and all the coated conditions after 168 h of immersion in Hanks’ solution are shown in Figure 10. The *R*_p_ values of the bare substrate remain in the same order of magnitude during the whole time of experimentation, but some fluctuations can be observed, especially between 48 and 96 h of immersion, where there is an increment in the *R*_p_ value. However, at the end of the experiment after 168 h of immersion, the final *R*_p_ value, 2.8 × 10^3^ Ω·cm^2^, is very close to the initial value after 1 h of immersion, 2.6 × 10^3^ Ω·cm^2^.

At the beginning of the test, the monolayer sol–gel coating systems SG and SG/GNP presented the highest *R*_p_ values, 6.4 × 10^6^ and 3.8 × 10^6^ Ω·cm^2^, respectively. The *R*_p_ value of the monolayer PEO coating was the lowest for all the systems, not only at the beginning of the tests but all along the experimentation time. Finally, the combination of sol–gel with PEO, as in the case of PEO + SG and PEO + SG/GNP, led to an increment of 1 order of magnitude in the *R*_p_ values of these coating systems, compared with the value of the monolayer PEO coating system.

At the end of the testing time, all the coating systems improved the behavior of the bare condition, with *R*_p_ values 1 order of magnitude higher than the values of the bare substrate, except for the PEO coating system, whose *R*_p_ value at the end of the experiment was only twice as high as the value of the bare substrate. On the other hand, the *R*_p_ values of the sol–gel monolayer coating systems SG and SG/GNP followed a downward trend throughout the experiment. However, the *R*_p_ values of the PEO, PEO + SG, and PEO + SG/GNP coating systems fluctuated until a certain stabilization was reached after 72 h of immersion.

The polarization resistance (*R*_p_) test showed that the monolayer SG and SG/GNP coatings behaved similarly, suffering an important decrease in their *R*_p_ values during the first 24 h, followed by a period of stabilization until 72 h, and then, the *R*_p_ values of both coating systems experienced a mild decrease until the end of the experiment. At the beginning of the experiment, the *R*_p_ values of these coatings were 3 orders of magnitude higher than the value of the bare substrate and at the end, only 1 order of magnitude higher. The *R*_p_ values of the combined PEO + SG and PEO + SG/GNP coating systems experienced a decreasing trend during the experimentation time but presented fluctuations. These fluctuations are related to the self-sealing effect of the porous PEO coatings.^57^ This effect is a consequence of the accumulation inside the pores of the hydrolysis products of the oxides and compounds of the coatings, sealing the pores and increasing the dielectric properties of the coating to some extent. For these samples, the *R*_p_ values were 2 orders of magnitude higher than the values of the bare substrate at the beginning of the experiment and 1 order of magnitude higher at the end. Finally, the monolayer PEO coating showed the lower *R*_p_ value of all the coating conditions for all the immersion times. In this case, the trend followed by the *R*_p_ values was descendent but suffered from fluctuations as in the case of the combined PEO/sol–gel coatings due to the same self-sealing effect.

**Figure 11:**
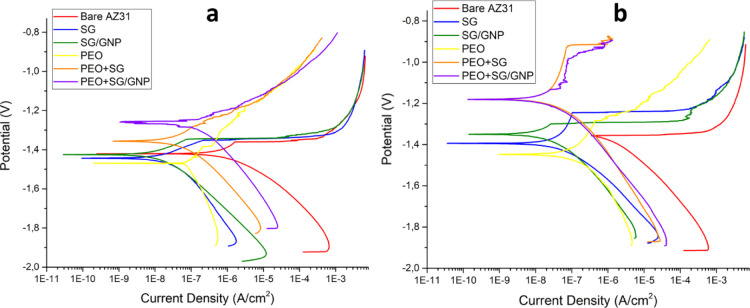
Anodic–cathodic polarization curves in Hanks’ solution of the different coating systems for (a) 1 and (b) 24 h of immersion.

The data from the anodic–cathodic tests carried out for 1 and 24 h of immersion in Hanks’ solution are shown in Figure 11 and Table 3.

**Table 3:** *E*_corr_ and Current Density Values from Tafel Tests after 1 and 24 h of Immersion

	1 h		24 h	
sample	*E*_corr_ (V)	*i* (A/cm^2^)	*E*_corr_ (V)	*i* (A/cm^2^)
Bare AZ31	–1.42	1.0·× 10^–6^	–1.36	1.5·× 10^–6^
SG	–1.44	1.8·× 10^–8^	–1.39	6.2·× 10^–8^
SG/GNP	–1.43	1.2·× 10^–8^	–1.35	2.4·× 10^–8^
PEO	–1.46	7.1·× 10^–8^	–1.45	1.6·× 10^–7^
PEO + SG	–1.36	3.1·× 10^–8^	–1.18	2.8·× 10^–8^
PEO + SG/GNP	–1.26	1.3·× 10^–7^	–1.18	2.5·× 10^–8^

After 1 h of immersion, the monolayer SG and SG/GNP coating systems presented current density values, which were 2 orders of magnitude lower than the value of the bare AZ31 substrate. The PEO + SG and PEO + SG/GNP coating systems presented less electronegative *E*_corr_ values and had current density values that were, respectively, 2 and 1 orders of magnitude lower than the value of the uncoated substrate but higher than the values of the monolayer SG and SG/GNP systems. Finally, the monolayer PEO coating presented the highest current density value of all the coated conditions but lower compared with the bare substrate. All coating systems achieved a great reduction in the current density compared with the bare substrate, probing their good behavior as protective coatings.

After 24 h of experiment, the current density value of the bare substrate slightly increased compared with the value after 1 h of immersion. Moreover, its *E*_corr_ value was the same as the pitting corrosion potential value. This phenomenon could be explained due to the NDE present in magnesium alloys, which can affect the results in electrochemical tests, inducing changes in the polarization curves, and in some cases creating linear regions, affecting the reaction around the corrosion potential.^52,58^ The current density values of the monolayer SG and SG/GNP systems slightly increased after 24 h of immersion. However, the current density values of the PEO and the combined PEO + SG and PEO + SG/GNP coating systems decreased. Pezzato et al.^59^ obtained similar results in their research. Moreover, similar current density values were obtained by Matykina et al.^60^ for monolayer PEO coatings generated from an electrolyte with the same composition as in our research but evaluated in SBF. PEO + SG and PEO + SG/GNP showed less electronegative *E*_corr_ values than all the other conditions.

For both immersion times, a passivation zone can be seen for monolayer sol–gel coatings and PEO coatings combined with a sol–gel coating. These passivation zones, which were present until the pitting corrosion potential was reached, are a result of the dielectric behavior of the different coating systems. For the bare AZ31, after 1 h of immersion, the oxide layer present on the surface of the sample provided some protection and, therefore, a passivation zone can be seen. However, after 24 h of immersion, the nonstable hydroxide layer present on the surface of the bare substrate cannot provide dielectric protection and, consequently, no passivation zone can be seen and the pitting potential is reached too fast. In the case of the monolayer sol–gel coatings, the dielectric protection is extended for the first 24 h of immersion as it can be extracted from the curves. The protective behavior of the PEO coatings changes for the different testing times. After 24 h of immersion, the PEO coatings combined with sol–gel show the lowest current density and the biggest passive behavior. This passivation behavior can be a consequence of the previously mentioned self-sealing effect of PEO coatings. Thus, the pores that could not be sealed by the applied sol–gel coating were filled with the electrolyte after 24 h of immersion. The deposition of the products generated by the hydrolysis of the oxides and the compounds of the coating, in contact with the electrolyte filling the pores, promotes the self-sealing effect, increasing the dielectric behavior of these coatings and decreasing the current density values.

The results obtained in the linear polarization resistance test matched those obtained in the anodic–cathodic polarization test. In this case, the current density values of all the coating conditions, except for the monolayer PEO coating, were close after both experimentation times. After 24 h of immersion, the current density values of these coating systems were 2 orders of magnitude lower than the values obtained for the bare substrate. The current density values of the monolayer PEO coating were the highest of all the coating conditions for both immersion times, 1 and 24 h, but both cases remained 1 order of magnitude lower than the values obtained for the bare substrate. Apart from current density values, a great difference was found between the corrosion potential for the monolayer PEO and the combined PEO/sol–gel coatings. For both immersion times, while the combined PEO + SG and PEO + SG/GNP coating systems presented the least electronegative *E*_corr_ values, the monolayer PEO coating showed the most electronegative *E*_corr_ values. The presence of a sol–gel layer that covers and fills the pores of the PEO coatings, as shown in Figures 3, 4, and 6, can explain this behavior, leading to an increment in the *R*_p_ values and a decrement in the current density values for the combined PEO/sol–gel coatings, compared with the monolayer PEO coating. The results obtained from the electrochemical tests suggest that the thickness values of the different PEO coatings, which were 1 order of magnitude higher than the values of the sol–gel coatings, did not seem to be a determinant factor to provide better protection than the monolayer sol–gel coatings. This could be a consequence of the fact that PEO coatings exhibit layered morphology with a porous outer layer and a dense inner layer. The outer layer is permeable to the aggressive media and therefore nonprotective against corrosion. The inner layer acts as a protective barrier. However, this inner layer presents thickness values lower than 1 μm, and it is usually not defect-free. That is the reason why, based on the electrochemical results, the protection provided by these coatings compared with the monolayer sol–gel coatings was not as high as expected regarding their thickness values. On the other hand, despite their lower thickness values, the monolayer sol–gel coatings were compact, crack-free, and well adhered to the substrates.

**Figure 12:**
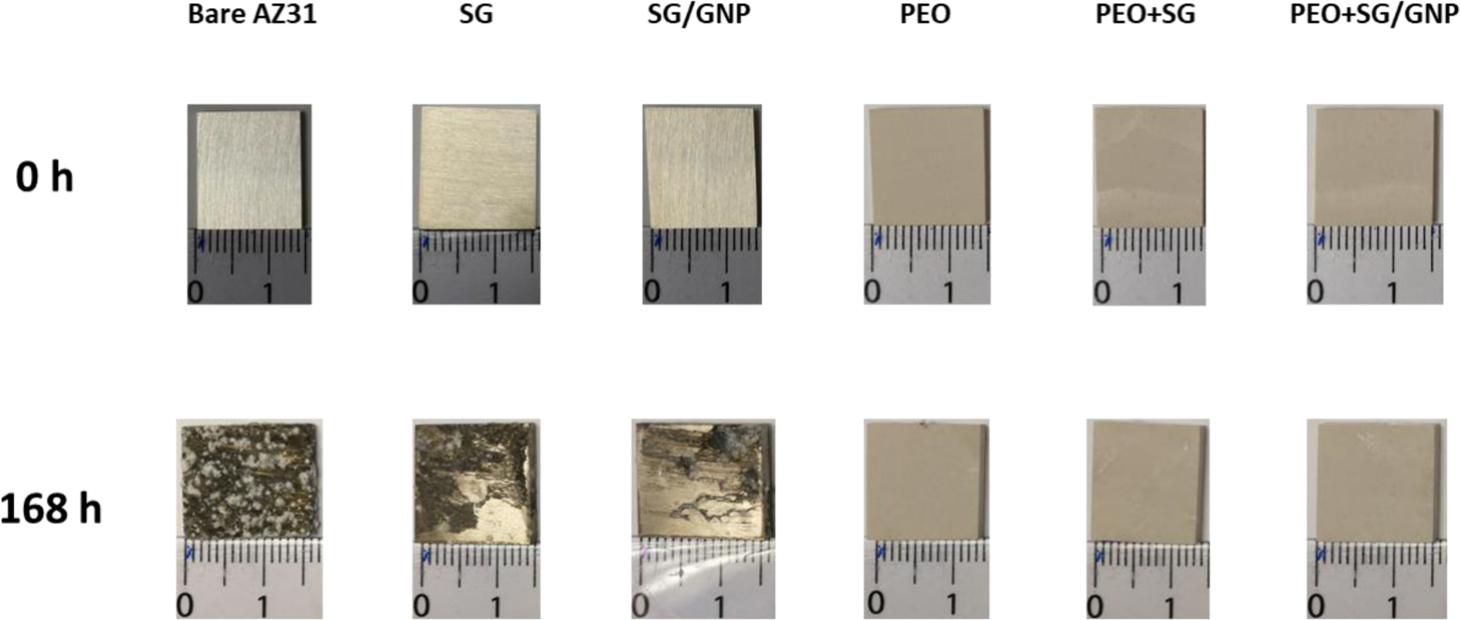
Visual assessment of the corrosion of AZ31 substrates coated with the different coating systems after immersion in Hanks’ solution for 1 week at 37 °C.

Figure 12 shows the progression of the corrosion process for a bare sample and one sample of each coating system, immersed for 168 h in Hanks’ solution at 37 °C.

For all the conditions, photographs were taken before and after 168 h of immersion to assess how the different coating systems behaved over this time. After 1 week of immersion in Hanks’ solution, the whole surface of the bare substrate was damaged and accumulations of corrosion products appeared distributed through the surface. The application of sol–gel monolayer coatings improved the behavior against corrosion. The surface of the substrates coated with SG and SG/GNP coating systems was slightly damaged, but at least half of the surface of the substrate coated with the SG coating was still protected and no corrosion signs were visible, and on the damaged zone, accumulation of corrosion products was observed. The protection was even higher for the substrate coated with the SG/GNP coating system, which after 168 h of immersion was barely affected, and the main damage was localized on the upper-right corner of the sample but without accumulation of corrosion products over the whole surface.

In the case of the PEO coatings after 1 week of immersion in Hanks’ solution, no corrosion or degradation signs were visible on the surface of both monolayer PEO coating and bilayer PEO + SG or PEO + SG/GNP coating systems. However, the electrochemical tests show that these samples suffered from corrosion processes after 168 h of immersion in the electrolyte. This apparent contradiction is caused by the fact that corrosion spreads between the PEO coatings and the AZ31 substrate in the coating-metal interface. The thickness and opacity of PEO coatings do not let to see the underlying damage; therefore, the samples appear intact on plain view images (Figure 12).

**Figure 13:**
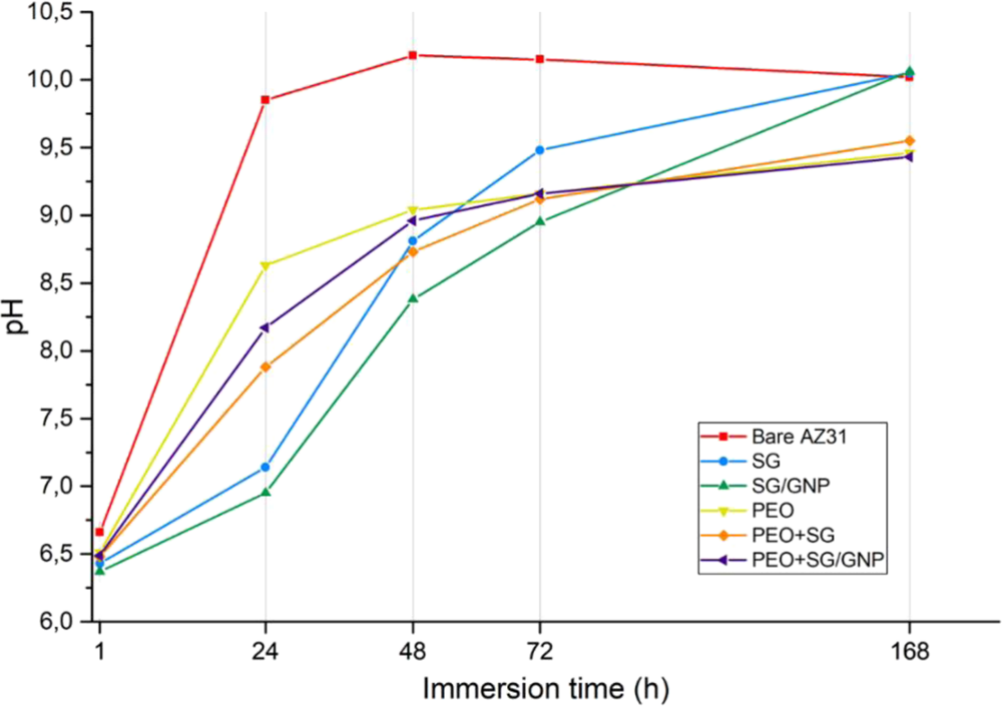
pH variation of Hanks’ solution for samples immersed at 37 °C for 1 week.

During the experimentation time, the pH value of the Hanks’ solution in which the samples shown in Figure 12 were immersed was measured every 24 h to assess its variation because some studies claim that pH variation of the fluid surrounding the implant can affect the corrosion rate of magnesium, increasing the corrosion rate for lower pH values.^9,61^ During the degradation of magnesium, H^+^ is consumed and OH^–^ is released, resulting in an increment of the pH value of the immersion medium, which favors the formation of a magnesium hydroxide film. This trend can be observed in Figure 13, where the evolution of the pH values of the Hanks’ solution in which the samples were immersed is shown.

During the first 24 h of immersion, the pH of the solution in which the bare substrate was immersed reached the highest value and faster than all the other conditions, reaching a value close to 10. After 24 h of immersion, the pH value tends to stabilize around 10 until the end of the experimentation time. In the case of the SG and SG/GNP coating systems, for the first 24 h of immersion, these coatings present the lowest pH values of all coating conditions, around 7. However, after 24 h of immersion, the pH values of these coating conditions increased faster until 168 h of immersion, when the pH reached the same value of the bare substrate. Finally, the pH values of PEO, PEO + SG, and PEO + SG/GNP coating systems behaved similarly. For the first 24 h of immersion, the pH of these coatings increased to higher values and faster than the pH values of the sol–gel monolayer coating conditions. However, after 24 h of immersion onward, this trend was inverted. After 168 h, the pH values of these PEO coatings were around 9.2, lower than the values of the monolayer sol–gel coatings and the bare substrate.

**Figure 14:**
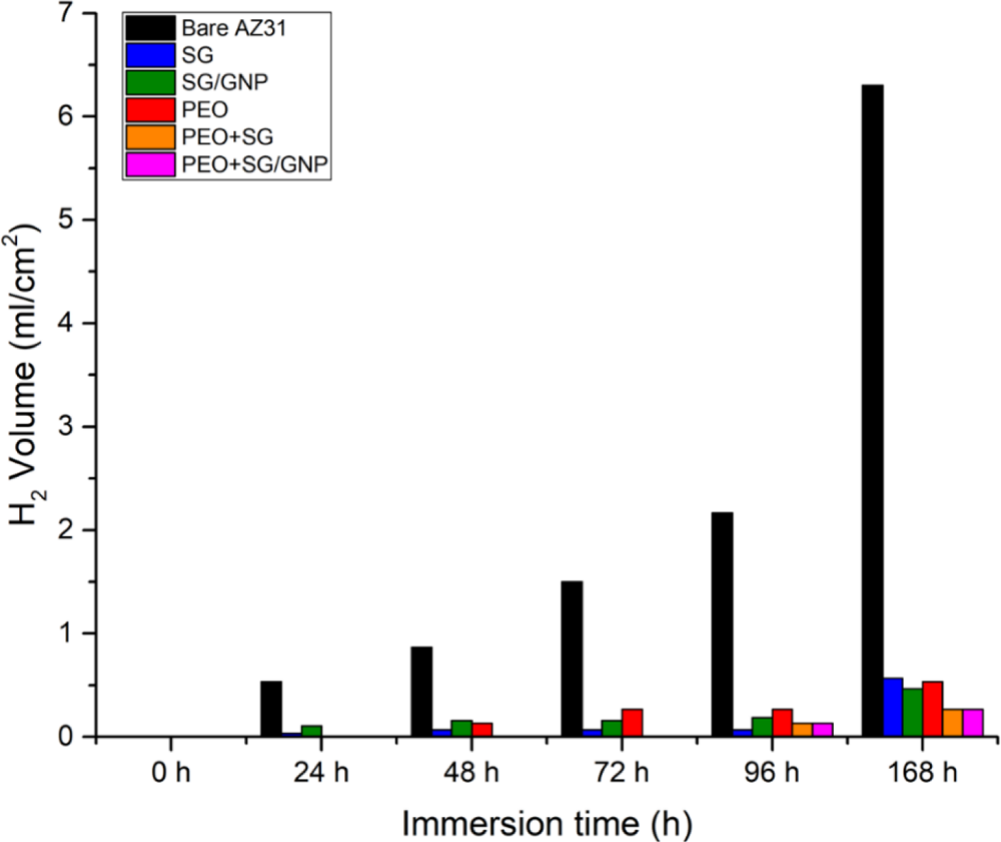
H_2_ evolution values for 168 h of immersion in Hanks’ solution.

As pointed out previously, the existence of the NDE in the corrosion of magnesium presents several problems for the reliable assessment of this material through electrochemical tests.^9,62^ Thus, the most simple and reliable technique to assess the in vitro degradation behavior of the different magnesium samples is the hydrogen evolution test. Figure 14 shows the results of the hydrogen evolution test carried out for 168 h of immersion of the different samples in Hanks’ solution at 37 °C. Differences between monolayer and bilayer coatings can be appreciated.

At the end of the experimentation time, low hydrogen volumes evolved from all the coated samples compared with the bare AZ31, especially in the case of the combined PEO + SG and PEO + SG/GNP systems.

Table 4 shows the values of the corrosion rate for each tested sample, calculated from the hydrogen evolution after 168 h of immersion in Hanks’ solution at 37 °C. The corrosion rate depends on the amount of evolved hydrogen. Therefore, lower corrosion rates were obtained for the samples where the hydrogen evolution was lower, the multilayer PEO + SG and PEO + SG/GNP coating systems. All the different coating configurations decrease the corrosion rate value of the coated substrates compared with the bare substrate. Moreover, the corrosion rate values obtained are lower than the values shown in the bibliography, obtained using SBF as corrosion medium for AZ31 substrates coated with two different coating systems; on the one hand, micro-arc oxidation coatings containing NaOH and KF and on the other hand, hydroxyapatite.^63^

**Table 4:** Corrosion Rate Calculated from Hydrogen Evolution after 168 h in Hanks’ Solution at 37 °C

sample	corrosion rate (mm/y)
Bare AZ31	2.05
SG	0.18
SG/GNP	0.15
PEO	0.17
PEO + SG	0.08
PEO + SG/GNP	0.08

**Figure 15:**
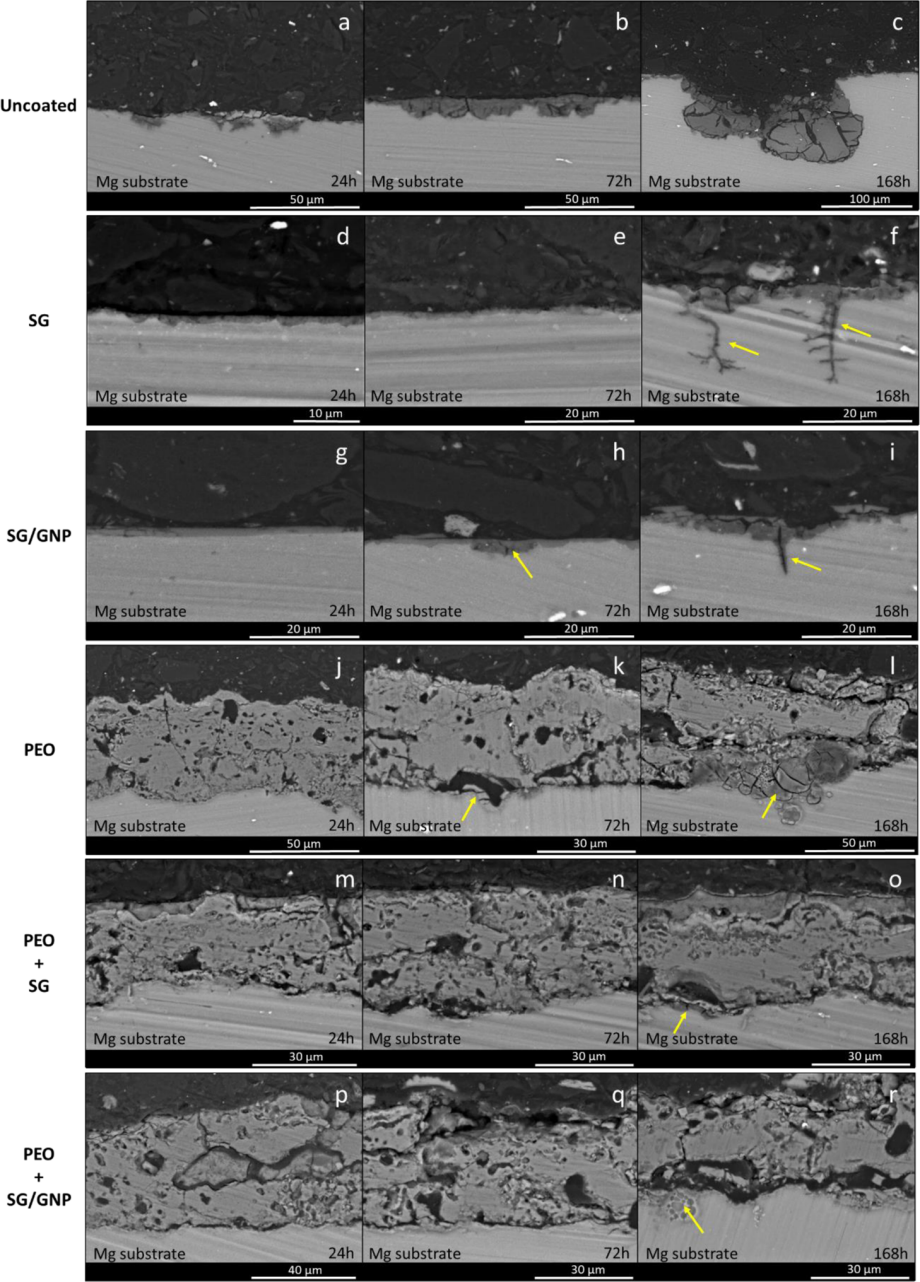
Cross section of micrographs of the different coating configurations after 24, 72, and 168 h of immersion in Hanks’ solution at 37 °C.

The results obtained in the hydrogen evolution test show that the protective behavior of the monolayer PEO and the combined PEO + SG and PEO + SG/GNP coatings was better than it was suggested from the electrochemical tests. In the case of the monolayer PEO coating, even though its *R*_p_ value after 168 h of immersion was close to that of the bare substrate, and the current density value was the highest for all the coating systems, the volume of evolved hydrogen was significantly lower than that for the bare substrate and close to that of the monolayer sol–gel coatings, which matches with the cross section of micrographs (Figure 15c,l). For the combined PEO + SG and PEO + SG/GNP coating systems, while their *R*_p_ and current density values were similar to that of the monolayer sol–gel systems, after 168 h of immersion in Hanks’ solution, the volume of evolved hydrogen was significantly lower in the case of the combined PEO + SG and PEO + SG/GNP coatings, and these results match with the micrographs shown in Figure 15o,r, where signs of corrosion are barely visible. The values of evolved hydrogen obtained in this test are close to the values shown in the bibliography for PEO coatings with the same composition.^42,60,64^ This research aims to control the degradation rate of the magnesium plates to decrease the generation of hydrogen and make this material compatible with biological environments and with the healing times of fractured bones, until their total degradation in the body. In this sense, the results obtained from the different corrosion tests indicate that combined PEO/sol–gel coatings provide the best degradation control, presenting the lowest corrosion rate values.

The visual assessment of the different coating systems after 168 h of immersion in the Hanks’ solution (Figure 12) showed that while the monolayer sol–gel coatings provided protection against corrosion to some extent and could be used to slow down the degradation rate of the magnesium substrates, the monolayer PEO and the combined PEO/sol–gel coatings provided the most effective protection against corrosion, and the magnesium substrates coated with these coating systems appeared to be unaffected. The evaluation of cross-sectional views of the different samples after 168 h of immersion could help to disclose the details of the corrosion mechanisms underlying the different coating systems.

Figure 15a–c shows the corrosion process of the bare substrate over the experimentation time. In this case, the uniform corrosion process began immediately after the immersion in Hank’s solution and covered the whole surface. During the experimentation time, the amount of corrosion products accumulated on the surface of the substrate increased, and at the end of the experiment, major damage could be observed. In the case of the monolayer SG and SG/GNP systems, evidence was found that the corrosion process started after 168 and 72 h of immersion, respectively (Figure 15f,h). The corrosion process started in the zones where the coating was damaged with the subsequent loss of protective capacity.

Once corrosion commences, it spreads under the coating due to a crevice corrosion process, accelerating the degradation of the coating and the loss of its protective capacity in other zones. At the end of the immersion time, in certain zones, the substrate under the coating was damaged and the coating was detached. Furthermore, intergranular corrosion appeared.

In the case of the monolayer PEO coating, although, as shown in Figure 12, no corrosion signs were visible on the surface of this sample, the assessment of the cross section of micrographs taken over 168 h of immersion showed the evolution of the corrosion process under the coating. After 24 h of immersion, no corrosion signs were visible (Figure 15j). After 72 h, the incipient corrosion of the substrate was detected in zones where the inner layer of the PEO coating was damaged (Figure 15k). At the end of the experimentation time, the electrolyte reached the interface between the PEO coating and the magnesium substrate through the interconnected pores. Due to the corrosion process, large accumulations of corrosion products were visible under the PEO coating in the zones where the inner layer was damaged. However, the opacity of this coating system made it impossible to appreciate the damage of the substrate, as shown in Figure 12. Finally, the combined PEO + SG and PEO + SG/GNP systems showed the best protective behavior. For the first 72 h of the experiment, no corrosion evidence was found for any of these coating systems (Figure 15n,q). At the end of the experimentation time, signs of corrosion were found, and slight accumulations of corrosion products were visible (Figure 15o,r).

The micrographs of Figure 15 show how the corrosion process evolves for the different samples. For the bare substrate, the corrosion is generalized and reaches high depth values. In the case of the monolayer SG and SG/GNP coatings systems, once the coatings were damaged due to the action of the aggressive species present in the medium, the electrolyte reaches the substrate and the corrosion starts. The corrosion spreads in the interface between the coating and the substrate (Figure 15h), cracking the coating and opening new pathways for the electrolyte to reach the substrate. Moreover, when the corrosion products grow enough, the coating detaches from the substrate, as shown in Figure 15f,i. In the case of the monolayer PEO coating, once the electrolyte has passed through the pores and reached the substrate, the corrosion process starts. As in the case of the monolayer sol–gel coatings, the corrosion products spread between the inner layer of the PEO coating and the underlying substrate (Figure 15l). The main difference between the monolayer PEO coating and the monolayer SG and SG/GNP coatings is that the PEO coating is much thicker than the sol–gel coatings. Furthermore, a bigger amount of corrosion products is necessary to detach this coating. After 168 h of immersion, the corrosion products do not grow enough to trigger the detachment of the coating. Finally, for the combined PEO + SG and PEO + SG/GNP coating systems, the presence of the outer sol–gel coating prevents the infiltration of the electrolyte through the pores of the underlying PEO coating, delaying the initiation of the corrosion process (Figure 15o,r).

However, the visual assessment of the samples shown in Figure 12 and the micrographs of the cross-sectional view shown in Figure 15 suggest that monolayer PEO and combined PEO + SG and PEO + SG/GNP coatings protected the magnesium substrates better than it could be deduced from the results of the electrochemical tests. This apparent inconsistency between the electrochemical tests and the results obtained by the visual assessment of the samples can be explained by the nature of the electrochemical tests and the morphology of the PEO coatings. Thus, the electrochemical tests measure the electrical resistance of the coatings, in the case of the polarization resistance tests, or the current density passing through the coatings, in the case of the anodic–cathodic tests. The thickness of the PEO coatings is high and so is their intrinsic porosity. The presence of interconnected pores and defects in the inner barrier layer can create direct pathways from the electrolyte to the substrate, which decreases the electrical resistance of the coatings or increases the current density passing through them. This means that the values obtained by electrochemical tests for the PEO coatings are not as good as expected from their thickness values or true surface area (considering porosity). However, the corrosion of the substrates coated with these coatings was not generalized and depends on the existence of a direct pathway created by interconnected pores and the existence of defects in the inner barrier layer. It is important to notice that in the case of combined PEO/sol–gel coatings, the presence of the sol–gel layer improves the polarization resistance and the current density values because sol–gel layers cause a decrease in the porosity of the underlying PEO coating, covering and filling part of the pores and decreasing the existence of direct pathways from the electrolyte to the magnesium substrate.

In the case of the monolayer sol–gel coatings, the lack of pores and defects provides them with better protective and isolating behavior, at least until the beginning of their degradation. However, once the monolayer sol–gel coatings start to degrade, generalized corrosion occurs on the substrates over which they were deposited. However, the higher thickness of the PEO coatings and their adhesion, derived from their genesis on the magnesium substrates, make them more durable. Therefore, for longer immersion times, the combined PEO/sol–gel coatings provide the best effective protection to the magnesium substrates.

### 3.3 Cytocompatibility Assessment

**Figure 16:**
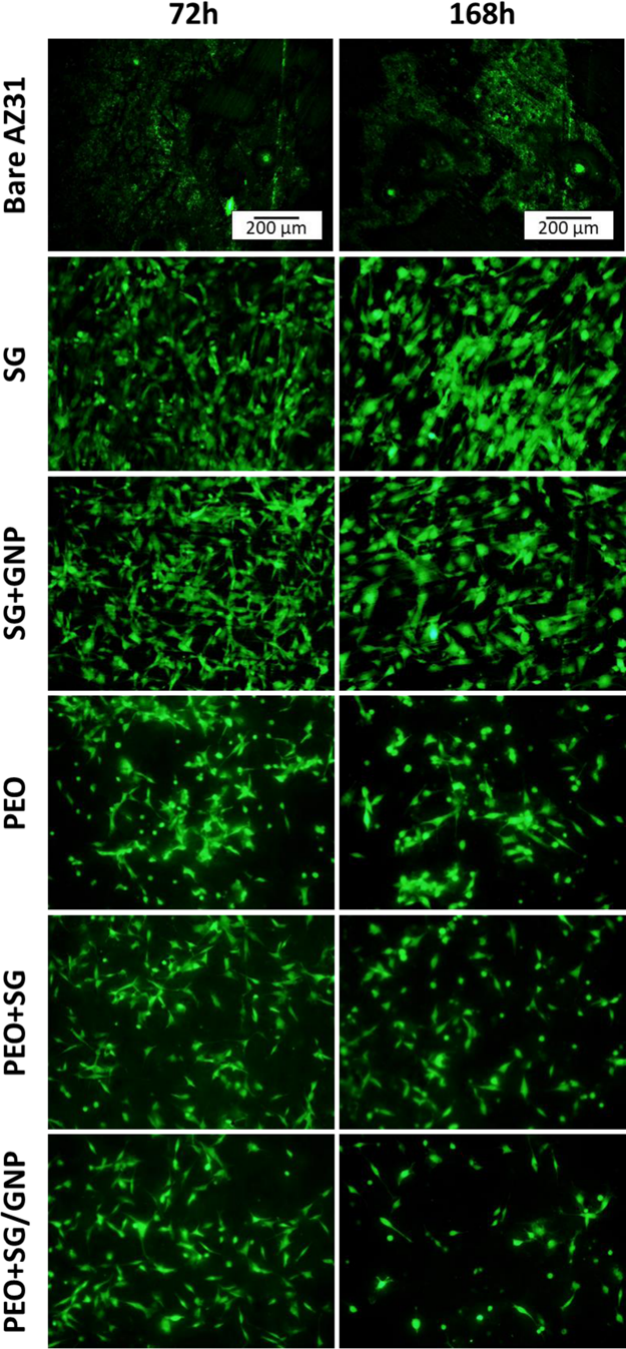
Fluorescence micrographs of the C2C12-GFP cell cultures on the surface of the different coating systems after 72 and 168 h of incubation.

After physicochemical characterization, the samples were evaluated to assess their cytocompatibility using the fluorescent C2C12-GFP premyoblastic cell line. Figure 16 shows fluorescence micrographs of the C2C12-GFP cell cultures growing on the surface of the different conditions after 72 and 168 h of incubation. In the case of the bare substrates, cells were not able to attach and grow on the surface due to the progress of the corrosion process at any moment of the incubation time. This can be due to the formation of corrosion products and the hydrogen evolution even at early immersion times. In the bibliography, examples can be found where the C2C12-GFP cell line was used to evaluate the cytocompatibility of magnesium alloys. A. Santos-Coquillat et al.^19^ seeded C2C12-GFP cells over Mg0.8Ca alloy substrates coated with different coating systems. After 120 h of study, the C2C12-GFP cells did not grow or proliferate over the bare Mg0.8Ca substrate, even though Mg and Ca are known to be essential elements in osteogenesis processes.^65^ The same behavior for the C2C12-GFP cell line was observed in the present research for the bare AZ31 alloy substrates.

After 72 and 168 h of incubation, all the coating systems improved the cytocompatibility of the samples. It was possible to observe higher cell confluence values in the case of the sol–gel monolayer coatings, that is, SG and SG/GNP coating systems. Similar behavior was described by Omar et al. for the same cell lineage seeded over sol–gel coatings with different compositions.^20^

In the case of the PEO coatings, that is, PEO, PEO + SG, and PEO + SG/GNP systems, lower cell proliferation was detected in comparison with SG and SG/GNP coatings. In addition, a visual assessment of the micrographs showed that there were slight differences between both incubation times. For these coatings, after 72 h of incubation, it was possible to observe an important number of nonadhered cells, especially in the PEO coating. After 168 h of incubation, the micrographs showed a slight decrease in the number of attached cells, and no monolayer was formed.

**Figure 17:**
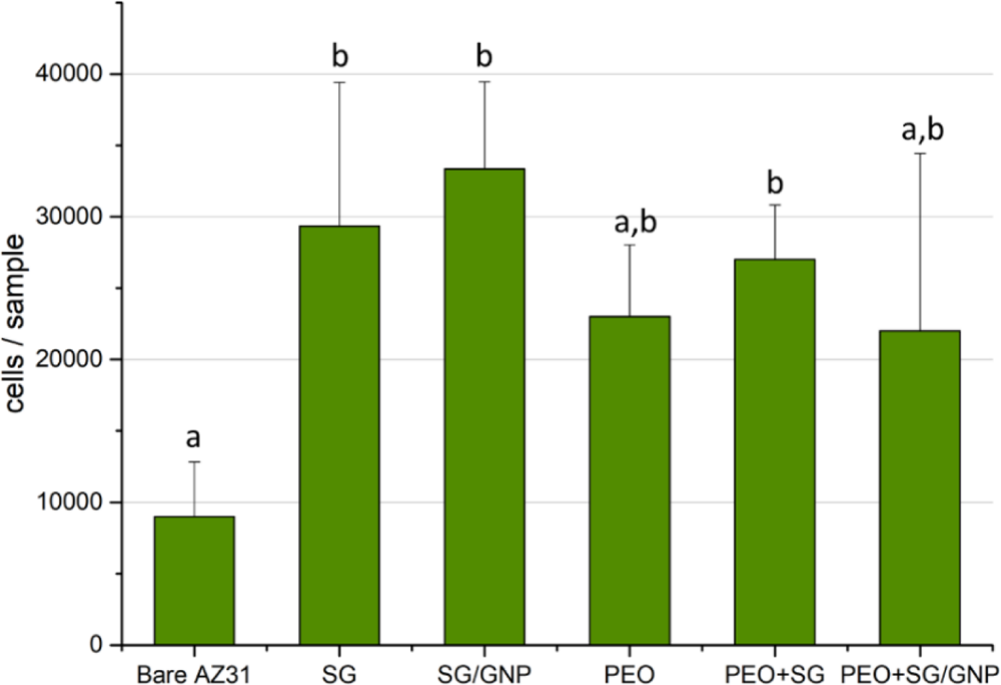
Viable cells on the different coating systems after 168 h of incubation. Bars bearing different letters present statistical differences (*p* < 0.05, ANOVA, Tukey’s test).

The cell density for the different coating conditions was assessed by detaching and counting the viable cells from the surface of the different samples. Figure 17 shows the number of viable cells at the end of the experiment. The general trend extracted from these data was that monolayer sol–gel coatings, that is, SG and SG/GNP systems presented higher cytocompatibility compared with all the PEO coatings, but no significant differences were visible between all coating systems. However, significant differences were observed between the bare substrate and the coated conditions.

**Figure 18:**
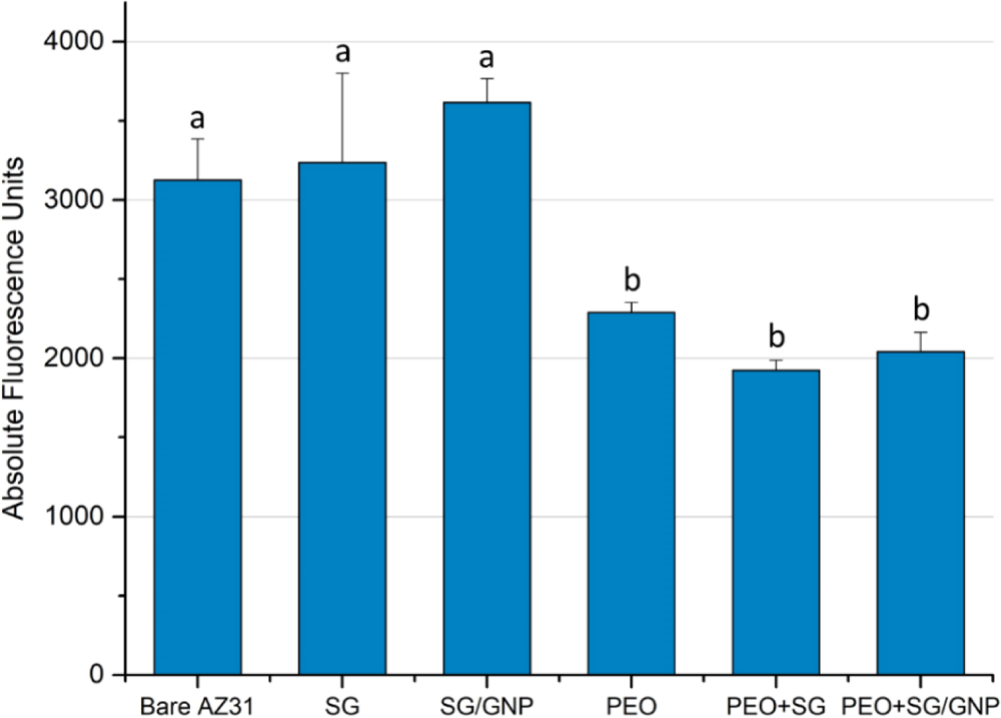
Metabolic activity values of the C2C12 cell cultures for the different coating systems after 168 h of incubation. Bars bearing different letters present statistical differences (*p* < 0.05, ANOVA, Tukey’s test).

The results of the metabolic activity tests after 168 h of incubation are shown in Figure 18.

The trend extracted from the data was that the cell growth was higher on the surface of the sol–gel monolayer coatings, SG and SG/GNP systems, which present the highest metabolic activity levels, with significant differences between these coating systems and the PEO, PEO + SG, and PEO + SG/GNP systems. In the case of the bare substrate, high fluorescence values were obtained. These values could be explained due to the presence of suspended clusters of the premyoblastic population proliferating in culture media.

**Figure 19:**
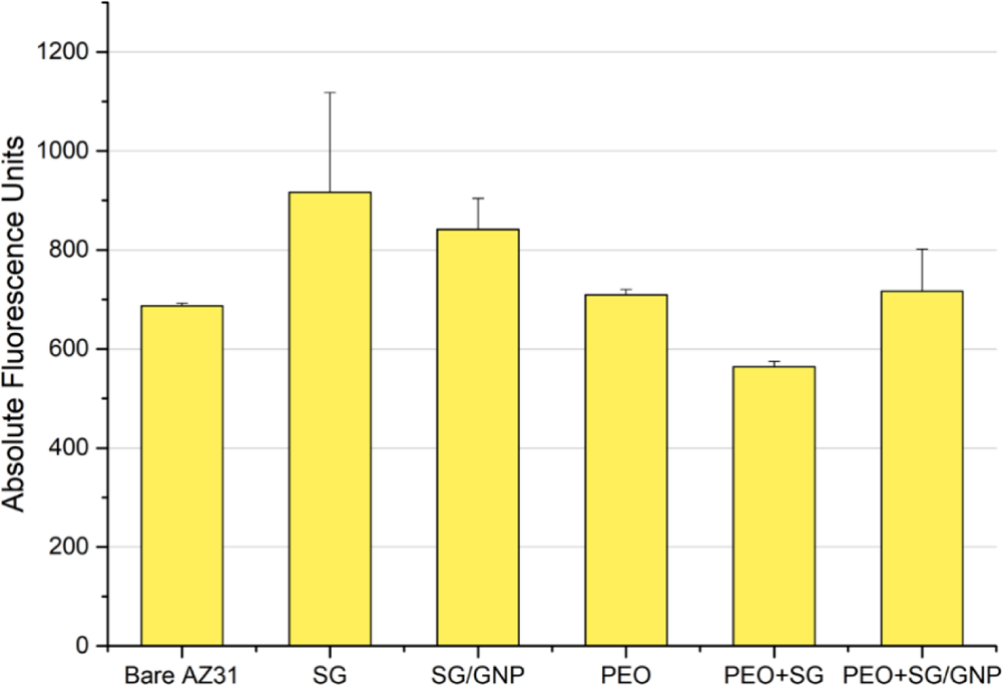
DNA quantitation values of the C2C12 cell cultures for the different coating systems after 168 h of incubation.

Hoechst staining was carried out to assess the performance of the C2C12 cells on the surfaces of the different coating configurations. Figure 19 shows the results for this test performed after 168 h of incubation.

In this case, as it happened in the previous cytocompatibility tests, the general trend was that the mean cytocompatibility values were higher in the case of the monolayer sol–gel coatings, that is, SG and SG/GNP systems. The SG coating without GNPs showed the highest mean DNA content. As in the case of the metabolic activity test, in the case of the bare substrate, high fluorescence values were obtained, showing that uncoated samples were not cytotoxic and enabled the proliferation of suspended cell clusters, but they did not allow cell adhesion and monolayer formation as it can be observed in the fluorescence micrographs for this sample.

Fluorescence micrographs show the evolution of the cell cultures. In the case of the bare AZ31 substrate, no cell proliferation was observed, viable but unattached cells were occasionally found in this sample. It is also important to highlight the presence of corrosion products spread all over the surface of the bare AZ31 substrate.

Looking at the micrographs of the monolayer sol–gel coatings, with and without GNPs, it was possible to observe good cell proliferation, and the cells were well attached to the surface of the coatings, showing an incipient formation of a cellular monolayer after 168 h of experiment. No significant differences were observed between the sol–gel coatings with or without GNPs. Similar results of cell adhesion and proliferation were found in the literature, where cell cultures of the same cell line (C2C12-GFP) were seeded on silica sol–gel coatings deposited over AZ91D magnesium substrates.^20^ In the case of the PEO coatings, for both monolayer and multilayer coating systems, it was possible to observe cell adhesion over surfaces. However, the presence of unattached cells was also detected. Compared with the monolayer sol–gel coatings, cell proliferation was much lower.

The cell growth over the different coating systems was evaluated using a Neubauer hemocytometer to count the viable cells after 168 h of incubation. The monolayer sol–gel coatings presented the highest number of cells and statistical differences with the bare AZ31 substrate. The monolayer and multilayer PEO coatings presented a lower number of cells compared with the monolayer sol–gel coatings. Only the value of the PEO + SG coating system was significantly higher than the bare substrate, and the PEO and PEO + SG/GNP coating systems presented higher mean values than the bare substrate, but no significant differences were found in the statistical analysis. In this study, the bare AZ31 substrate presented the lowest mean values of cell growth. The direct count of viable cells in the hemocytometer prevented false readings due to the previous elimination of the medium and, therefore, the presence of clusters of detached cells. Thus, these values for the different coating systems were all consistent with what was observed in the fluorescence micrographs.

The metabolic activity of the cell cultures on the different coating systems was evaluated using alamarBlue staining after 168 h of incubation. The highest fluorescence values, and therefore the highest metabolic activity, corresponded to the monolayer sol–gel coatings, without significant differences between the two types. The PEO coatings, both the monolayer and the multilayer combined with a sol–gel coating, show the lowest fluorescence values and present statistical differences with the monolayer sol–gel coating systems. These results are consistent with the fluorescence micrographs. However, in the case of the bare AZ31 substrate, the fluorescence value was as high as for the monolayer sol–gel coatings. However, no cell proliferation was observed on the surface of these samples in the fluorescence micrographs, and this inconsistency could be explained by the presence of suspended clusters of the premyoblastic population proliferating in culture media, which can cause an unspecific signal and false readings in the microplate reader used for the evaluation of the metabolic activity.

DNA quantification of the cell cultures over the different coating systems was evaluated using Hoechst staining after 168 h of incubation. As in the case of the metabolic activity test, the highest mean fluorescence values, and therefore the highest DNA quantification, corresponded to the monolayer sol–gel coatings. The fluorescence values for all the PEO coatings were still lower but closer to the values of the monolayer sol–gel coatings. However, no statistical differences were found in the ANOVA analysis for this test. These results for the different coating systems are consistent with the fluorescence micrographs. However, as in the metabolic activity study, the fluorescence value for the bare AZ31 substrate was, in this case, as high as for the PEO coatings. Again, no cell proliferation was observed in the fluorescence micrographs for this sample, and as previously exposed, this inconsistency could be explained by the presence of suspended clusters of cells proliferating in culture media, causing false readings in the microplate reader.

The cytocompatibility behavior of the PEO coatings was lower than expected. The presence of fluorine in the PEO composition could be responsible for this behavior. Fluorine, at some concentrations, has been described in the literature to be cytotoxic.^66−68^ In previous research, a PEO coating generated from an electrolyte with a NaF concentration of 8 g/L showed cytotoxicity due to the high release of F^–^,^19^ which could be the same case as in the present research. The monolayer sol–gel coatings showed the best improvement, with the highest values of cellular growth and adhesion. Also, in the viable cell counting, metabolic activity, and DNA quantitation tests, these coating configurations obtained the best cytocompatibility results.

The sol–gel synthesis method raises interest in the generation of biomaterials that can be used for different applications in biomedicine. For example, research can be found in the scientific literature where silica or hydroxyapatite materials generated by sol–gel are used to create scaffolds for their use in tissue engineering and bone fracture treatments,^69^ as well as porous matrices, used to create bioartificial organs by enclosing cells inside the porous material, which allows for the exchange of metabolites between the cells and the physiological medium.^70^ The use of the sol–gel process for the generation of drug delivery systems has been also reported in the literature.^71−74^

Another advantage of sol–gel coatings is the possibility to generate hierarchical coatings. For example, one layer in contact with the substrate to protect it against corrosion processes, followed by a second bioactive layer to improve the biocompatibility or a biocide layer to prevent infections once implanted in the body. Hierarchical coatings can also be developed by the combination of different coatings methods. Examples of that are presented in this research, in the case of the PEO + SG and PEO + SG/GNP coating systems. Both sol–gel and PEO coatings allow for the addition of different compounds and particles during the coating generation process to improve the existing or provide new properties. For example, PEO coatings could be loaded with corrosion inhibitors to improve corrosion resistance. Over this first PEO coating, a sol–gel coating doped with growth factors or antibiotics could be deposited to improve the biological properties of the coating system. There is a wide range of possibilities to generate multifunctional hierarchical coatings, and more research in this field is worth it.

## 4 Conclusions

All the coating configurations decreased the corrosion rate of the magnesium alloy immersed in simulated physiological medium. The nature of the different coating systems influenced their performance during the corrosion tests.

The SG and SG/GNP coatings provide good protection at short immersion times. The PEO coatings were intrinsically porous, facilitating the electrolyte to reach the underlying substrate. The PEO + SG and PEO + SG/GNP coatings presented the highest corrosion protection. Big differences were found between these systems and the monolayer PEO coating, proving that the sol–gel coating effectively sealed the pores of the PEO and improved the corrosion protection.

The SG and SG/GNP coatings showed the highest cytocompatibility for the studied cell line in all the tests. The PEO coatings showed lower cytocompatibility than expected. The presence of fluorine in their composition, cytotoxic at certain concentrations, could affect this behavior.
